# The Role of Diet in Bone and Mineral Metabolism and Secondary Hyperparathyroidism

**DOI:** 10.3390/nu13072328

**Published:** 2021-07-07

**Authors:** Matteo Bargagli, Maria Arena, Alessandro Naticchia, Giovanni Gambaro, Sandro Mazzaferro, Daniel Fuster, Pietro Manuel Ferraro

**Affiliations:** 1 U.O.C. Nefrologia, Dipartimento di Scienze Mediche e Chirurgiche, Fondazione Policlinico Universitario A. Gemelli IRCCS, 00168 Roma, Italy; maria.arena90@virgilio.it (M.A.); alessandro.naticchia@policlinicogemelli.it (A.N.); 2 Dipartimento Universitario di Medicina e Chirurgia Traslazionale, Università Cattolica del Sacro Cuore, 00168 Roma, Italy; 3 U.O.C. Nefrologia, Azienda Ospedaliera Universitaria Integrata di Verona, 37126 Verona, Italy; giovanni.gambaro@univr.it; 4 Department of Translational and Precision Medicine, Sapienza University of Rome, 00161 Rome, Italy; sandro.mazzaferro@uniroma1.it; 5 Nephrology Unit, Policlinico Umberto I, 00161 Rome, Italy; 6 Department of Nephrology and Hypertension, Inselspital, Bern University Hospital, University of Bern, 3010 Bern, Switzerland; daniel.fuster@dbmr.unibe.ch

**Keywords:** bone and mineral metabolism, secondary hyperparathyroidism, chronic kidney disease, dietary advice

## Abstract

Bone disorders are a common complication of chronic kidney disease (CKD), obesity and gut malabsorption. Secondary hyperparathyroidism (SHPT) is defined as an appropriate increase in parathyroid hormone (PTH) secretion, driven by either reduced serum calcium or increased phosphate concentrations, due to an underlying condition. The available evidence on the effects of dietary advice on secondary hyperparathyroidism confirms the benefit of a diet characterized by decreased phosphate intake, avoiding low calcium and vitamin D consumption (recommended intakes 1000–1200 mg/day and 400–800 UI/day, respectively). In addition, low protein intake in CKD patients is associated with a better control of SHPT risk factors, although its strength in avoiding hyperphosphatemia and the resulting outcomes are debated, mostly for dialyzed patients. Ultimately, a consensus on the effect of dietary acid loads in the prevention of SHPT is still lacking. In conclusion, a reasonable approach for reducing the risk for secondary hyperparathyroidism is to individualize dietary manipulation based on existing risk factors and concomitant medical conditions. More studies are needed to evaluate long-term outcomes of a balanced diet on the management and prevention of secondary hyperparathyroidism in at-risk patients at.

## 1. Introduction

Bone and mineral metabolism is characterized by a complex interaction between minerals and hormones such as calcium, phosphate and magnesium, parathyroid hormone (PTH), fibroblast growth factor-23 (FGF23) and active vitamin D (1,25(OH)_2_D_3_). Bone disorders are a common complication of chronic kidney disease (CKD) and dietary advice plays a fundamental role in its management [1,2]. Here, we review the physiology and pathophysiology of bone and mineral metabolism and the effect of diet in its regulation.

## 2. Methods

In this article, we reviewed the available literature regarding the association between diet and all causes of secondary hyperparathyroidism. We searched articles from accessible online databases (PubMed, the Cochrane library, and Web of Science). The articles of interest were obtained using the following search terms: (“diet” OR “dietary advice” OR “foods” OR “nutrition”) AND (“secondary hyperparathyroidism” OR “mineral and bone disorder” OR “CKD-MBD”). In this review, only articles written in English language and with available full text were included. A total of 198 references were included in this review.

## 3. Physiology of Bone and Mineral Metabolism

Bone is a specialized connective tissue hardened by apatite, a form of calcium phosphate. The homeostasis of bone is regulated by three different kind of cells: osteoclasts, osteoblasts and osteocytes [3,4]. Osteoblasts are responsible for bone formation and secretion of non-mineralized bone matrix [5]. The activation of PTH, 1,25(OH)_2_D_3_, growth hormone (GH) and estrogen receptors on osteoblasts surface leads to the production of insulin-like growth factor 1 (IGF1), a crucial autocrine hormone that drives postnatal skeletal growth [3]. Osteoclasts are indirectly stimulated by PTH via osteoblasts to release calcium phosphate from bone [5] and are regulated by the osteoprotegerin/receptor activator nuclear receptor ligand (OPG/RANK-L) system [6]: osteoblasts express RANKL, which binds RANK on the surface of osteoclast precursors, inducing their maturation. When the decoy receptor OPG is secreted by osteoblasts, it binds RANK-L with higher affinity than RANK [7], preventing excessive bone resorption [8]. By contrast, decreased OPG secretion increases stimulation of osteoclasts [6]. Besides, osteocytes, the differentiated form of osteoblasts [9], secrete several hormones (e.g., FGF23 and sclerostin) and express matrix proteins that regulate bone–mineral remodeling [9].

Almost 99% of total body calcium is stored in bones, with a calcium/phosphate ratio of 2:1 [3]. The total calcium balance is regulated by PTH, 1,25(OH)_2_D_3_, and calcitonin through their interaction with the kidneys, bones and gastrointestinal tract [10]: a reduction in serum calcium induces PTH and 1,25(OH)_2_D secretion, increasing both its renal retention and intestinal absorption, whereas hypercalcemia is counterbalanced by calcitonin secretion and PTH inhibition. Besides, recent research points to discoveries about the links between bone cells and bone marrow cells, whose interactions might help explain the occurrence of bone disease in inflammatory states [11].

Intestinal calcium uptake is either paracellular or transcellular, and the latter is positively regulated by calcitriol activation [12].

The kidneys fine tune the serum calcium concentration by increasing or decreasing its urinary excretion. Calcium reabsorption mainly occurs by the paracellular route in the proximal tubule and thick ascending limb (TAL) driven by active sodium reabsorption, whereas in the distal convoluted tubule (DCT) calcium reabsorption is transcellular and regulated by PTH, calcitonin and 1,25(OH)_2_D_3_ [13]. Furthermore, activation of the calcium-sensing receptor (CaSR) inhibits calcium transport along the TAL through upregulation of claudin-14, which likely blocks divalent cation permeable pores formed by claudin-16 and claudin-19 in this segment [14,15].

PTH, 1,25(OH)_2_D_3_, FGF23 and Klotho are also involved in maintaining a constant serum phosphate concentration. Phosphate is mainly stored in bone [16] and is transported transcellularly by Na-Pi cotransporters, in either the small intestine (NpT2b, SLC34A2) or the proximal tubule (NpT2a and NpT2c, SLC34A1 and SLC34A3, respectively). PTH exerts its phosphaturic function by reducing NpT2a and NpT2c activity [16], but it decreases the expression of NpT2b [17], as well. Moreover, low serum phosphate levels increase 1,25(OH)_2_D_3_ synthesis [18], whereas hyperphosphatemia inhibits 1α-hydroxylase exacerbating 1,25(OH)_2_D_3_ deficiency [19].

Both increased dietary phosphate intake and calcitriol stimulate FGF23 secretion [20,21]. However, the activation of the Fibroblast Growth Factor Receptor 1 by FGF23 requires a cofactor named Klotho, a kidney-derived hormone. FGF23 reduces the expression of tubular sodium/phosphate cotransporters [22] and inhibits the synthesis of 1,25(OH)_2_D_3_ by downregulation of 1α-hydroxylase [23] and increases the degradation of 1,25(OH)_2_D_3_ by stimulating its degrading enzyme 24-hydroxylase [24,25]. In addition, FGF23 acts on parathyroid tissue to decrease PTH secretion [26].

## 4. Pathophysiology of Secondary Hyperparathyroidism

**Figure 1:**
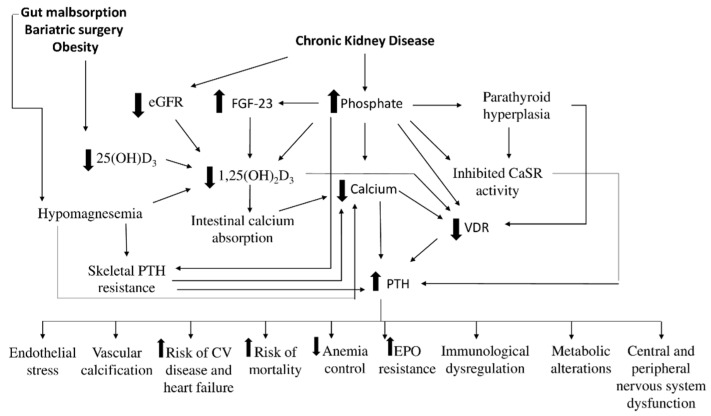
Mechanisms of secondary hyperparathyroidism.

Secondary hyperparathyroidism (SHPT) is defined as (appropriately) increased PTH secretion due to an underlying stimulus. The pathophysiological primum movens of the increased PTH secretion is either reduced serum calcium or increased serum phosphate concentrations [27]. The most frequent conditions associated with SHPT include gut malabsorption, renal failure, or other causes of vitamin D deficiency (Figure 1) [27,28]. Reduced vitamin D concentration is commonly found in obese patients, although the underlying mechanism is not completely understood: reduced outdoor physical activity and vitamin D sequestration in adipose tissue have been hypothesized [29,30]. Apart from the origin of vitamin D deficiency, an inverse association between BMI and serum 25(OH)D_3_ concentrations was found [31,32,33]. Bariatric surgery [34] may also lead to vitamin D deficiency of multifactorial origin: obese patients are at higher risk for vitamin D insufficiency and the surgical procedure may predispose these patients to malabsorption syndrome [35,36]. Gut malabsorption is also a known complication of celiac disease and inflammatory bowel diseases such as Crohn’s disease [37,38,39]. Conversely, reduced intestinal uptake of calcium alone is not enough to cause SHPT, unless accompanied by severe vitamin D deficiency or additional risk factors such as renal failure [40].

Of note, hypomagnesemia, a described complication of gut malabsorption [41], may worsen SHPT by inducing hypocalcemia, low serum 1,25(OH)_2_D_3_ and impaired PTH secretion and increased skeletal PTH resistance [42].

Secondary hyperparathyroidism and mineral and bone disorders are some of the most common complications of chronic kidney disease (CKD-MBD), even in early stages [43,44].

During CKD progression, phosphate is progressively retained with a subsequent reduction in serum calcium, due to the formation and deposition of calcium-phosphate salts [45]. This stimulates PTH secretion to restore serum calcium concentration and reduce serum phosphate through its phosphaturic effect on renal tubules. An additional effect of hyperphosphatemia is the inhibition of 1,25(OH)_2_D_3_ synthesis, either directly, or indirectly by increasing plasma FGF23 concentration [46,47]. Furthermore, decreased glomerular filtration rate (GFR) and low serum 25(OH)D_3_ limit the synthesis of active vitamin D in these patients [48]. Besides, both reduced active vitamin D concentration and inhibited CaSR activity driven by raised serum phosphate insufficiently suppress PTH secretion [49,50,51,52]. Hyperphosphatemia also stimulates progressive parathyroid hyperplasia with a subsequent reduction in the expression of vitamin D and calcium receptors, predisposing patients to a dysregulation of the parathyroid cells’ response to circulating calcium and calcitriol [21]. Moreover, either hypocalcemia, hyperphosphatemia or decreased serum calcitriol could lead to a reduction in VDR expression, worsening 1,25(OH)_2_D_3_ resistance on parathyroid cells [53].

Increased circulating plasma FGF23 concentrations are already present in early CKD stages, long before the occurrence of hyperphosphatemia [54,55]. Another player in the field is represented by Klotho, the coreceptor of FGF23, responsible for its selective target organ effects. Circulating soluble alpha-Klotho levels are reduced very early in CKD and it is thus possible that increments of FGF23 represent a compensatory response of osteocytes to reduced peripheral response [56]. Increased plasma FGF23 is associated with increased mortality [57,58,59] and poor cardiovascular outcomes [60,61,62,63].

Similarly, PTH levels start to increase at normal or near normal GFR levels, long before the onset of hypocalcemia or hyperphosphatemia [44]. Whatever the cause, once secondary hyperparathyroidism develops, it increases the risk for several conditions. Elevated PTH levels are associated with endothelial stress, vascular calcification, increased risk for cardiovascular events and heart failure and increased mortality [64]. SHPT is also associated with poor anemia control and resistance to erythropoietin therapy in CKD patients [65], peripheral and central nervous system and immunological dysfunction, glucose and lipid metabolic alterations [66].

Ultimately, osteocytes and osteoclasts seem to be involved in the function of a new anatomical microenvironment of hematopoietic stem cells (HSCs), known as the bone marrow niche [67]. These provide support to HSCs maturation, proliferation, and mobilization. Recently, chronic inflammatory diseases such as diabetes and CKD have been hypothesized to induce bone niche dysfunctions [11]. In fact, PTH, vitamin D, FGF23 and CaSR all have a role in niche regulation and a model of CKD-MBD found a significant reduction in HSCs in bone marrow, coupled with higher immature colony-forming units in the spleen and peripheral blood [68].

## 5. Effect of Diet on Bone and Mineral Metabolism Homeostasis and Secondary Hyperparathyroidism

### 5.1. Dietary Protein Intake

Bone health is influenced by several nutrients and food components that are normally ingested daily, through different mechanisms, including: endocrine and/or paracrine effects, actions on bone turnover and the homeostasis of calcium, phosphate and magnesium [69].

Several studies investigated the role of dietary protein intake on bone mineral density (BMD), bone mineral content (BMC) and the risk of fracture. The data from randomized controlled trials (RCTs) are contradictory: some authors demonstrated a positive relationship between protein intake and BMD in different bone segments [70,71,72,73], while others found no association between protein intake and bone mass [74,75,76,77,78,79,80]. Cohort studies revealed similar conflicting results [81,82,83,84,85]. A possible explanation for the association may be related to IGF-I secretion: Schürch et al. [73] demonstrated that increased protein intake causes a rise in circulating IGF-I, which exerts an anabolic effect on bone (Table 4). At the same time, low serum IGF-I was associated with reduced BMD and cortical bone thickness in a rat model of low protein diet [86,87]. The source of dietary protein seems to be equally relevant for bone health: a low-protein, soy-based diet significantly decreases femoral cortical thickness, compared to a low-protein, casein-oriented diet, in mice [88].

With respect to bone and mineral biomarkers, a number of clinical trials analyzed the effect of dietary protein consumption on osteocalcin concentration, with conflicting results: Uenishi et al. [89] and Bharadwaj et al. [90] found higher levels in patients with increased protein intake, whereas others observed no differences in osteocalcin concentrations [71,72,73,76,91,92]. Furthermore, Kerstetter et al. [76] reported that in case of low protein consumption, the intestinal absorption of calcium is reduced with a consequent increase in circulating PTH and calcitriol. The intake of dietary protein also seems to be inversely associated with PTH secretion [93], possibly indicating a beneficial effect of a protein-oriented diet.

### 5.2. Dietary Acid and Alkali Intake

Since the 1920s it has been known that there is a positive association of protein intake with urinary calcium excretion [94] and both renal and extrarenal mechanisms seem to be involved [95]. A possible explanation for this phenomenon relates to the acid load induced by dietary protein. Meat and fish are rich in sulfur-containing amino acids, generating a fixed metabolic acid load. On the contrary, fruits and vegetables provide more alkali than acids [96]. Acid retention stimulates bone resorption by activating osteoclasts and inhibiting osteoblasts [97]. In addition, acid loading increases urinary calcium excretion due to altered tubular calcium handling. These changes are independent of PTH, 1,25(OH)_2_D_3_ and tubular sodium handling [98,99]. As a consequence, dietary acid loads were found to be associated with a negative calcium balance and low BMD [93,97,98,100,101]. Recent data, however, suggest that acid-load-independent mechanisms may also be involved in the increase in urinary calcium by dietary protein [102], including higher filtered load and reduced fractional tubular reabsorption of calcium due to increased GFR or augmented intestinal calcium uptake, especially in cases of high protein and low calcium intake [103,104].

Renal net acid excretion from diet can be estimated, using a validated formula, through determination of the potential renal acid load of foods (PRAL) (Table 1) [105]. Cao et al. [93] found that a diet characterized by higher PRAL increased both urinary calcium excretion and its intestinal absorption (Table 4).

The alkaline diet theory affirms that a diet rich in fruit and vegetables would be essential for maintaining bone integrity, although the data regarding this hypothesis are controversial [106]. Frassetto et al. [107] and MacDonald et al. [108] provided no evidence for such a theory, whereas RCT data [109] supported it: 60 mEq of potassium citrate supplementation significantly increased BMD after 24 months, in 201 elderly patients without CKD.

A Mediterranean diet, which is rich in legumes and vegetables, was indeed associated with higher bone mineral density [110], an effect possibly mediated by the high alkali and low acid content of the diet [111,112,113]. However, an alkali-oriented diet is also associated with increased magnesium intake. In fact, magnesium deficiency has been linked to impaired bone growth, osteoblastic and osteoclastic activity, osteopenia, bone fragility and altered calcium metabolism [42]. Few studies also suggested that magnesium supplementation is associated with increased BMD and reduced bone turnover [114,115,116].

### 5.3. Calcium Intake

Peak bone mass in early adulthood strongly depends on dietary calcium intake and skeletal calcium retention [117,118]. Several studies demonstrated that calcium consumption significantly predicts total body bone mass, and subjects with reduced calcium intake may not achieve their target peak bone mass [119,120,121]. Moreover, a Japanese study reported a positive association between calcium intake and bone density in young adults [122] and higher dietary calcium improved BMC and BMD [123]. Thus, the amount of dietary calcium (Table 2), along with its interaction with vitamin D, seems to play an important role in bone and mineral homeostasis. In fact, Spiegel et al. [124] found an inverse relationship between dietary calcium content, 1,25(OH)_2_D_3_ and PTH levels (Table 4). Despite a reduction in circulating 1,25(OH)_2_D_3_, net intestinal calcium absorption was found to be increased with a high calcium diet, probably through augmented paracellular absorption [125]. Conversely, in case of a low calcium diet, the 1,25(OH)_2_D_3_-dependent transcellular intestinal uptake of calcium prevails [69]. Although both vitamin D and animal-derived calcium intake have been inversely correlated with PTH [126], some other authors observed a null effect of total dietary calcium on the risk for SHPT [40]. This led to the hypothesis that calcium bioavailability (and hence the impact on PTH) varies by dietary source. Accordingly, Gannagé-Yared et al. [126] confirmed the abovementioned inverse association between PTH and dairy and animal sources of calcium, whereas vegetable-derived calcium caused no changes in plasma PTH concentrations [127]. The latter may be at least partially explained by the high oxalate content of plant-derived foods, which is known to decrease intestinal calcium absorption [126].

In view of these results, one may expect a deleterious effect on bone health of a diet in which vegetables and fruits are the only source of calcium. However, it is well known that such a diet is associated with higher BMD [128]. The underlying mechanisms are currently not clear and may be multifactorial [129]. Certainly, the reduced acid and increased magnesium content of a diet rich in vegetables and fruits has also to be taken into account. By contrast, a strict vegetarian diet is associated with lower 25(OH)D_3_ levels and characterized by low dietary calcium intake and an increased PTH concentration [130].

### 5.4. Phosphate Intake

Phosphate is an essential nutrient in the human diet (Table 3) and plays an important role in intracellular signaling and energy metabolism and is a key constituent of cell membranes and bone. The available literature clearly demonstrates that a high dietary phosphate intake increases plasma PTH (Table 4) [131,132,133,134,135,136,137]. Accordingly, Hayakawa et al. [138] found parathyroid cell proliferation and increased PTH secretion in an animal model with high dietary phosphate intake. In fact, the initial phosphaturic response after either an intestinal or intravenous phosphate load occurred after the increase in circulating PTH, whereas the rise in circulating FGF23 and the fall in 1,25(OH)_2_D_3_ were noted later [139].

A diet rich in phosphate and low in calcium increased plasma PTH in younger adults [131], although there was no difference compared with an isolated low calcium diet [140]. In addition, Katsumata et al. [141] found that if an elevated dietary phosphate intake is accompanied by high calcium intake, it blunts the effect of the increased phosphate load on PTH secretion. Two large surveys showed no relationship between dietary phosphate intake and BMD or BMC, confirming the beneficial effects of a diet rich in calcium or with a high calcium/phosphate ratio on bone [142,143]. In contrast, however, the abovementioned studies observed a direct association between low phosphate intake and decreased BMD [142,143].

### 5.5. Lipid Consumption

Obesity is known to alter vitamin D metabolism by reducing circulating 25(OH)D_3_ through an incompletely understood mechanism [144]. Mice fed with a diet rich in saturated fatty acids showed reduced 25(OH)D_3_ and increased 1,25(OH)_2_D_3_ concentrations, compared to a low-fat diet. This was caused by induction of renal 1α-hydroxylase, inhibition of 24-hydroxylase and decreased expression of 25-hydroxylase mRNA in the liver and elevated plasma PTH. Thus, the authors hypothesized that a high fat diet might induce a state of increased PTH secretion, leading to altered 1,25(OH)_2_D_3_ regulation, partially explaining the reduced activity of hepatic 25-hydroxylase in obese patients [145].

On the contrary, a greater consumption of monounsaturated fatty acids, such as olive oil, has been found to improve bone health [146,147]. They contain phenolic compounds that can positively modulate the proliferation and maturation of osteoblastic cells by increasing alkaline phosphatase activity and calcium ions deposition in the extracellular matrix [148]. Tyrosol and hydroxytyrosol, two of the main phenolic compounds of olive oil, have been found to improve the loss of bone mass induced by inflammation [149].

Finally, recent evidence suggests a positive effect of weight loss on 25(OH)D_3_ concentrations, demonstrating a direct association between serum vitamin D and weight reduction [150,151,152], especially when the weight loss was between 5% and 10% [150,151].

## 6. Diet and Secondary Hyperparathyroidism in Non-Dialysis-Dependent CKD

The mainstay of CKD-related secondary hyperparathyroidism treatment is the correction of the pathogenic triggers known to induce or worsen this condition. Hyperphosphatemia is indeed the most common risk factor associated with SHPT in case of reduced renal function [153]. First-line therapy therefore aims at avoiding hyperphosphatemia. Historically, the most common dietary advice was to reduce protein intake, especially consumption of animal-derived foods [154]. In the last decade, dietary advice for protein restriction changed, focusing not on the absolute intake of phosphates but rather on the sources of the phosphates ingested [155,156].

The KDOQI nutritional guidelines 2020 [1] suggested that metabolically stable patients affected by CKD stage G3–G5 should start a very low protein diet (0.55–0.6 or 0.28–0.43 g/kg/day with keto-acid/amino acid analogues) to reduce CKD progression and death. However, only a few RCTs have investigated the effect of such a diet on nutritional parameters including serum albumin, total protein or muscle strength [157,158] and a risk of sarcopenia and malnutrition might exist, especially for older patients.

In addition the updated KDIGO guideline on CKD-MBD 2017 suggests maintaining serum phosphate in the reference range in patients affected by CKD stage G3a-G5 by reducing dietary phosphate intake or starting a phosphate binder [159].

RCTs with clinical outcomes on phosphate binders showed that the obtained reduction in circulating phosphate was indeed accompanied by lower mortality for both dialyzed and non-dialyzed CKD patients [160,161], whereas calcium-free phosphate binders seem to also reduce the progression of vascular calcifications [162]. On the contrary, the efficacy of dietary measures on plasma phosphate is controversial: some authors find only a modest or null effect of dietary phosphate restriction in lowering serum phosphate [160,163,164,165], which does not necessarily reflect total phosphate balance and reduced phosphate intake may be counterbalanced by a compensatory decrease in FGF23 and PTH concentration. To confirm these mechanisms, Ritter and colleagues found a direct association between phosphate restriction and calcium-sensing receptor (CaSR) expression and function in a uremic rat model. Switching from a 1.2% to a 0.2% phosphate diet led to normalization of CaSR expression, arrested parathyroid hyperplasia and reduced PTH [166]. At the same time, it was reported that phosphate restriction elicits a fall in circulating FGF23 concentrations in CKD patients [167].

An analysis of patients with estimated GFR < 60 mL/min/1.73 m^2^ and available 24 h dietary data in the Third National Health and Nutrition Examination Survey [168], revealed that participants in either the highest or the lowest quartile for dietary phosphate intake had no differences in serum phosphate concentrations. In addition, in patients not receiving maintenance hemodialysis, phosphate intake was not associated with all-cause mortality.

More recently, Selamet et al. [169] observed that dietary phosphate intake, estimated through 24 h urinary phosphate excretion in 795 patients with CKD stage G3a-G5, was not associated with higher risk of end-stage kidney disease and all-cause mortality. The authors raised a concern regarding excessive dietary protein restriction and the inherent risk for malnutrition, which is already known to increase the risk of death in CKD patients [170].

These results may lead to the conclusion that dietary advice yields only a modest effect on the management of SHPT. However, the use of urinary phosphate excretion alone may be not a strong predictor of phosphate balance in patients affected by CKD [171]: in patients with normal renal function, a dietary phosphate load does not lead to hyperphosphatemia because of a compensatory increase in urinary phosphate excretion induced by phosphaturic hormones such as PTH and FGF-23 [167,172]. In contrast, in patients with advanced CKD, this response is impaired, possibly increasing the risk of a positive phosphate balance, even if salivary and gastrointestinal phosphate clearances are increased [173,174,175].

At the same time, significant differences between direct determination of phosphate contents in foods compared with nutritional databases were recently reported [155]. This inaccuracy, together with hidden dietary phosphate sources, raises further concern in predicting the average intake of phosphate in CKD patients by dietary recalls and it may at least partially explain the heterogeneous results in the literature on the effects of phosphate restriction. The utility of the urinary phosphate/urea nitrogen ratio in the identification of patients with higher inorganic phosphate intake was recently demonstrated [176]. This may be used as a useful tool for monitoring hidden dietary phosphate sources in CKD patients.

The relationship between the phosphate content of foods and its gastrointestinal absorption is even more complex. In fact, the source of dietary phosphate has been demonstrated to modify the risk of hyperphosphatemia as well. This was shown in a crossover trial including nine patients with CKD stages G3-G4, who switched from a meat-based diet to a purely vegetarian diet [155]. Although the total dietary intakes of phosphate (795 vs. 810 mg/day, respectively) and calcium (1176 vs. 1310 mg/day, respectively) were similar, the patients consuming only vegetarian foods showed reduced plasma phosphate and FGF23 concentrations and a slight increase in PTH, compared to the meat-based diet.

An analysis of 2918 participants in the Chronic Renal Insufficiency Cohort (CRIC) [177] with available dietary data confirmed the former results: higher intake of plant vs. meat proteins was associated with reduced plasma FGF23 and increased serum bicarbonate concentrations, without affecting serum phosphate or plasma PTH levels.

The source of dietary phosphate likely explains these results: the bioavailability of inorganic phosphate, which is commonly used as a food preservative and in processed meat, is high [156,178,179]. Despite the total amount of phosphate being similar between meat and vegetables, the organic phosphate found in vegetables such as legumes, nuts and cereals is mainly present in form of phytate [180]. Since humans lack the phytate degrading enzyme phytase, the bioavailability of phosphate in plants is reduced compared with animal proteins.

The linkage between altered acid-base homeostasis and SHPT in CKD patients is well characterized and a state of metabolic acidosis was found to be associated with worsening mineral bone disease by directly increasing bone buffering, PTH and FGF23 secretion [181,182,183], leading to negative calcium balance and loss of bone mineral density [97,184].

Recently, dietary acid load was also taken into account in the regulation of phosphate homeostasis. Higher dietary acid load has been associated with reduced plasma bicarbonate in patients affected by CKD [185]. This was shown to be counterbalanced by higher serum phosphate and urinary phosphate excretion, in both cases probably due to a compensatory increase in titratable acid excretion and bone buffering [185,186].

In CKD patients, serum calcium concentration is normal until the most advanced stages of the disease, when it may decrease. Whereas the development of hypocalcemia is a further stimulation for PTH secretion, excessive positive calcium balance may increase the risk for ectopic calcifications and cardiovascular risk. The available evidence on dietary calcium [124,187] show that a balanced calcium intake of approximately 800–1000 mg/day decreases plasma PTH without affecting serum calcium or phosphate concentrations in non-dialyzed CKD patients who are not taking active vitamin D. These considerations are in line with the average recommended calcium consumption for healthy subjects [188].

## 7. Diet and Secondary Hyperparathyroidism in Dialysis-Dependent CKD

The nutritional requirements of CKD patients change substantially when they start renal replacement therapy [1,189]. As previously described for CKD patients, increased plasma PTH and hyperphosphatemia have been found to be a frequent and deleterious condition associated with the progression of mineral bone disease and an increased risk for all-cause mortality [190,191,192]. Furthermore, it was estimated that CKD-MBD accounts for up to 20% of overall mortality in hemodialysis patients [193]. Besides, in those patients there is also a high risk of malnutrition, which itself is linked to higher mortality rates. Hence, the latest nutritional guidelines on CKD [1] recommend avoidance of a low protein diet in both hemodialysis and peritoneal dialysis patients and advocate a daily dietary protein intake between 1.0 and 1.2 g/kg body weight. Instead of pursuing a low protein diet, it was demonstrated that a useful method for reducing phosphate intake is to counsel hemodialysis patients on identifying foods containing phosphate additives, leading to a significant serum phosphate decrease after 3 months [194].

Compared to non-dialysis dependent CKD, the maintenance of a neutral calcium balance is much more complex in hemodialysis patients: it is influenced by calcium consumption, active vitamin D supplementation, calcium content in the dialysate and dialysis modality [195]. It was proposed that if total calcium intake exceeds 1500 mg/day, especially in patients receiving active vitamin D analogues [196], it would increase the risk for extraosseous calcifications [197].

## 8. Conclusions

The available evidence on dietary manipulation in the management of secondary hyperparathyroidism confirms the beneficial effect of a diet characterized by lower phosphate and balanced calcium (800–1000 and 1000–1200 mg/day for CKD patients and the general population, respectively) [119,123] and vitamin D (400–800 IU/day) [198] content, especially in a subset of medical conditions such as CKD, obesity and gut malabsorption. Besides, reduced protein intake in CKD patients is associated with better control of SHPT risk factors, although its strength in maintaining serum phosphate in the reference range and the resulting outcomes are debated, mostly for dialyzed patients. In addition, consensus on the effect of dietary acid loads on bone health has not been reached. In conclusion, a reasonable approach for reducing the risk for secondary hyperparathyroidism is to individualize dietary manipulation based on existing risk factors and concomitant medical conditions. More studies are needed to evaluate long-term outcomes of a balanced diet on the management and prevention of secondary hyperparathyroidism in at-risk patients.

## Figures and Tables

**Table 1:** Foods with high PRAL (PRAL in mEq per 100 g serving).

Cereal and Grain	Fish and Shellfish	Meat and Poultry	Others
**Wheat (12.26)**	Tuna (9.19–12.71)	Chicken (8.73–13.84)	Leavening agents (270.16)
**Rye (11.95)**	Salmon (11.11)	Veal (4.96–12.64)	Sweets (0.38–85.40)
**Couscous (7.60)**	Mackerel (5.13–8.42)	Beef (4.83–12.53)	Cheese (6.18–21.29)
**Rice (6.98)**	Crab (8.37)	Pork (3.99–12.44)	Eggs (9.42)
**Macaroni (6.93)**	Lobster (7.44)	Turkey (7.37–10.49)	Nuts (−16.04; 8.71)
**Breakfast cereals (0.23)**	Cod (6.53)	Lamb (4.41)	

Data from the US Department for Agriculture (USDA).

**Table 2:** Foods with high calcium content (calcium content in mg per 100 g serving).

Dairy	Animal-Derived	Vegetables	Others
**Milk (276 mg)**	Sardines (286 mg)	Lamb’s quarters (362 mg)	Tahini (902 mg)
**Kefir (247 mg)**	Salmon (179–212 mg)	Nettles (334 mg)	Almond milk (345 mg)
**Buttermilk (222 mg)**		Amaranth (216 mg)	Rice milk (221 mg)
**Yogurt (216 mg)**		Spinach (191 mg)	
**Cheese (138–333 mg)**		Soybeans (175 mg)	
**Greek yogurt (116 mg)**		Kale (94 mg)	

Data from the US Department for Agriculture (USDA 2002) and Ferraro et al. [100].

**Table 3:** Foods with high phosphate content (phosphate content per 100 g serving).

Dairy Products	Meat and Fish	Legumes and Vegetables	Others
**Cheese (464–602 mg)**	Canned sardines (489 mg)	Soybeans (180 mg)	Sesame seeds (616 mg)
**Cottage cheese (116–143 mg)**	Canned salmon (326 mg)	Mushrooms (140 mg)	Walnuts (510 mg)
**Yogurt, all types (89–141 mg)**	Pork (173–294 mg)	Chickpeas (130 mg)	Pistachio nuts (500 mg)
**Milk (87–110 mg)**	Veal (237–258 mg)	Beans (103 mg)	Almonds (440 mg)
	Beef (178–231 mg)	Cabbage (70 mg)	Curry powder (260 mg)
	Tuna (138 mg)	Broccoli (60 mg)	Peanuts (250 mg)

Data from the Harvard T.H. Chan School of Public Health Nutrition Department.

**Table 4:** Dietary factors associated with an increased risk of SHPT.

Dietary Factors	Potential Effect on Bone and Mineral Metabolism
**High protein intake**	Increased IGF-I and osteocalcin
**High dietary acid load**	Increased urinary calcium excretion
**Low calcium intake**	Increased 1,25(OH)_2_D_3_ and PTH
**Insufficient vitamin D supplementation**	Reduced renal reabsorption and intestinal uptake of calcium
**Low fruits and vegetables intake**	Potential decrease in BMD
**Low magnesium intake**	Potential osteopenia and negative calcium balance
**Higher phosphate intake**	Increased FGF23 and PTH
